# *catena*-Poly[[bis­(4-methyl­benzoato-κ^2^ *O*:*O*′)lead(II)]-μ-nicotinamide-κ^2^ *N* ^1^:*O*]

**DOI:** 10.1107/S1600536810028126

**Published:** 2010-07-17

**Authors:** Tuncer Hökelek, Hakan Dal, Barış Tercan, Efdal Çimen, Hacali Necefoğlu

**Affiliations:** a Department of Physics, Hacettepe University, 06800 Beytepe, Ankara, Turkey; b Department of Chemistry, Faculty of Science, Anadolu University, 26470 Yenibağlar, Eskişehir, Turkey; c Department of Physics, Karabük University, 78050 Karabük, Turkey; d Department of Chemistry, Kafkas University, 36100 Kars, Turkey

## Abstract

In the title compound, [Pb(C_8_H_7_O_2_)_2_(C_6_H_6_N_2_O)]_*n*_, the Pb^II^ ion is coordinated by two 4-methyl­benzoate (PMB) and one nicotinamide (NA) ligands while symmetry-related NA ligands bridge adjacent Pb^II^ ions, forming polymeric chains along the *c* axis. The carboxyl­ate groups in the two PMB ions are twisted away from the attached benzene ring by 22.9 (2) and 4.6 (2)°. The two benzene rings of the PMB ions are oriented at a dihedral angle of 83.7 (1)°. In a polymeric chain, the NA ligands are linked to PMB ions through intra­molecular N—H⋯O hydrogen bonds. In the crystal structure, adjacent polymeric chains inter­act *via* N—H⋯O and C—H⋯O hydrogen bonds, forming a two-dimensional network parallel to the *bc* plane.

## Related literature

For niacin, see: Krishnamachari (1974 ▶) and for *N*,*N*-diethyl­nicotinamide, see: Bigoli *et al.* (1972 ▶). For related structures, see: Greenaway *et al.* (1984 ▶); Hökelek & Necefoğlu (1996 ▶); Hökelek *et al.* (2009*a* ▶,*b* ▶,*c* ▶,*d* ▶).
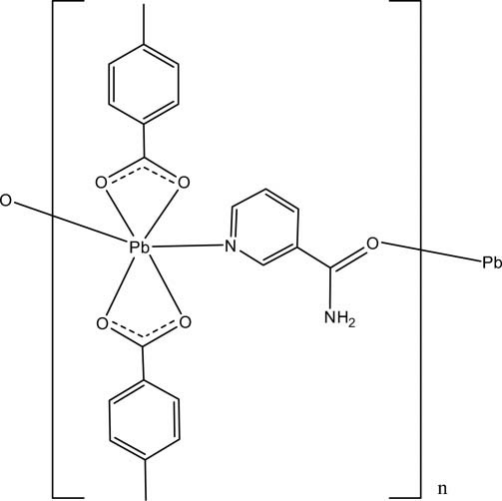


## Experimental

### 

#### Crystal data

[Pb(C_8_H_7_O_2_)_2_(C_6_H_6_N_2_O)]
*M* *_r_* = 599.60
Monoclinic, 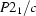

*a* = 14.1146 (3) Å
*b* = 7.7431 (2) Å
*c* = 19.2165 (4) Å
β = 102.322 (2)°
*V* = 2051.81 (8) Å^3^
*Z* = 4
Mo *K*α radiation
μ = 8.26 mm^−1^
*T* = 100 K
0.34 × 0.32 × 0.13 mm

#### Data collection

Bruker Kappa APEXII CCD area-detector diffractometer
Absorption correction: multi-scan (*SADABS*; Bruker, 2005 ▶) *T*_min_ = 0.074, *T*_max_ = 0.342
19461 measured reflections
5143 independent reflections
4669 reflections with *I* > 2σ(*I*)
*R*_int_ = 0.030

#### Refinement

*R*[*F* ^2^ > 2σ(*F* ^2^)] = 0.020
*wR*(*F* ^2^) = 0.050
*S* = 1.03
5143 reflections
281 parameters
H atoms treated by a mixture of independent and constrained refinement
Δρ_max_ = 1.08 e Å^−3^
Δρ_min_ = −1.03 e Å^−3^

### 

Data collection: *APEX2* (Bruker, 2007 ▶); cell refinement: *SAINT* (Bruker, 2007 ▶); data reduction: *SAINT*; program(s) used to solve structure: *SHELXS97* (Sheldrick, 2008 ▶); program(s) used to refine structure: *SHELXL97* (Sheldrick, 2008 ▶); molecular graphics: *Mercury* (Macrae *et al.*, 2006 ▶) and *ORTEP-3 for Windows* (Farrugia, 1997 ▶); software used to prepare material for publication: *WinGX* (Farrugia, 1999 ▶) and *PLATON* (Spek, 2009 ▶).

## Supplementary Material

Crystal structure: contains datablocks I, global. DOI: 10.1107/S1600536810028126/ci5133sup1.cif

Structure factors: contains datablocks I. DOI: 10.1107/S1600536810028126/ci5133Isup2.hkl

Additional supplementary materials:  crystallographic information; 3D view; checkCIF report

## Figures and Tables

**Table 1:** Selected bond lengths (Å)

Pb1—O1	2.7594 (19)
Pb1—O2	2.3141 (17)
Pb1—O3	2.4824 (18)
Pb1—O4	2.5672 (19)
Pb1—O5	2.6800 (16)
Pb1—N1^i^	2.661 (2)

Symmetry code: (i) 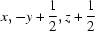.

**Table 2:** Hydrogen-bond geometry (Å, °)

*D*—H⋯*A*	*D*—H	H⋯*A*	*D*⋯*A*	*D*—H⋯*A*
N2—H2*A*⋯O3	0.86 (3)	2.02 (3)	2.835 (3)	158 (3)
N2—H2*B*⋯O2^ii^	0.86 (3)	2.11 (3)	2.946 (3)	167 (3)
C4—H4⋯O1^iii^	0.93	2.53	3.431 (3)	165
C11—H11⋯O1	0.93	2.59	3.253 (3)	129
C17—H17⋯O2^ii^	0.93	2.41	3.317 (3)	166

Symmetry codes: (ii) 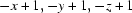; (iii) 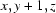.

**Fig. 1.**
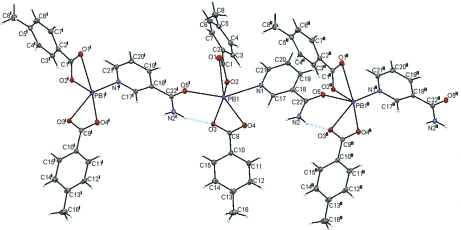
Part of the polymeric chain of the title compound, with the atom-numbering scheme. Displacement ellipsoids are drawn at the 50% probability level. Primed atoms are generated by the symmetry operators: (') x, 1/2 - y, 1/2 + z; ('') x, 1/2 - y, z - 1/2. Dashed lines indicate hydrogen-bonding.

**Fig. 2.**
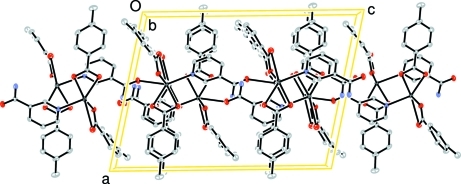
The crystal structure of the title complex.

## supplementary crystallographic information

### Comment

As a part of our ongoing investigation on transition metal complexes of nicotinamide (NA), one form of niacin (Krishnamachari, 1974), and/or the nicotinic acid derivative *N*,*N*-diethylnicotinamide (DENA), an important respiratory stimulant (Bigoli *et al.*, 1972), the title compound was synthesized and its crystal structure is reported herein.

In the crystal structure of the title compound, each Pb^II^ ion is coordinated by two 4-methylbenzoate (PMB) and one nicotinamide (NA) ligands (Fig. 1), while symmetry related NA ligands bridge the Pb^II^ ions forming polymeric chains along the *c* axis (Fig. 2). The two PMB ions act as bidentate ligands, while the NA is monodentate ligand (Fig. 1). The crystal structures of similar complexes of Cd^II^, Co^II^, Mn^II^ and Zn^II^ ions, [Cd(C_8_H_5_O_3_)_2_(C_6_H_6_N_2_O)_2_(H_2_O)].H_2_O, (II) (Hökelek *et al.*, 2009*a*), [Co(C_9_H_10_NO_2_)_2_(C_6_H_6_N_2_O)(H_2_O)_2_], (III) (Hökelek *et al.*, 2009*b*), [Mn(C_9_H_10_NO_2_)_2_(C_6_H_6_N_2_O)(H_2_O)_2_], (IV) (Hökelek *et al.*, 2009*c*), [Zn_2_(DENA)_2_(C_7_H_5_O_3_)_4_].2H_2_O, (V) (Hökelek & Necefoğlu, 1996) and [Zn(C_8_H_8_NO_2_)_2_(C_6_H_6_N_2_O)_2_].H_2_O, (VI) (Hökelek *et al.*, 2009*d*) have also been reported. In (II), the two benzoate ions are coordinated to the Cd atom as bidentate ligands. In the other structures one of the benzoate ligands acts as a bidentate ligand, while the other is monodentate ligand.

The average Pb—O bond length (Table 1) is 2.5606 (18) Å and the Pb1 atom is displaced out of the least-squares planes of the carboxylate groups (O1/C1/O2) and (O3/C9/O4) by -0.096 (10) Å and 0.403 (10) Å, respectively. The O1/C1/O2 and O3/C9/O4 carboxylate planes form dihedral angles of 22.9 (2)° and 4.6 (2)°, respectively, with benzene rings A(C2-C7) and B(C10-C15), while the angles between rings A, B and C (N1/C17-C21) are A/B = 83.7 (1), A/C = 65.4 (1) and B/C = 20.9 (1)°. An intramolecular N—H···O hydrogen bond (Table 2) links the NA ligand to one of the carboxylate groups of the PMB ions acting as a bidentate ligand. In (I), the O1—Pb1—O2 and O3—Pb1—O4 angles are 51.09 (6)° and 51.71 (5)°, respectively. The corresponding O—M—O (where M is a metal) angles are 52.91 (4)° and 53.96 (4)° in (II), 60.70 (4)° in (III), 58.45 (9)° in (IV), 58.3 (3)° in (V), 60.03 (6)° in (VI) and 55.2 (1)° in [Cu(Asp)_2_(py)_2_] (where Asp is acetylsalicylate and py is pyridine) [(VII); Greenaway *et al.*, 1984].

In the crystal structure, N—H···O and C—H···O hydrogen bonds (Table 2) link adjacent chains into a two-dimensional network parallel to the *bc* plane (Fig.2).

### Experimental

The title compound was prepared by the reaction of Pb(NO_3_)_2_ (1.656 g, 5 mmol) in H_2_O (50 ml) and nicotinamide (1.220 g, 10 mmol) in H_2_O (10 ml) with sodium 4-methylbenzoate (1.580 g, 10 mmol) in H_2_O (160 ml). The mixture was filtered and set aside to crystallize at ambient temperature for four weeks, giving colourless single crystals.

### Refinement

Atoms H2A and H2B of the NH_2_ group were located in a difference Fourier map and refined isotropically. The remaining H atoms were positioned geometrically with C–H = 0.93 and 0.96 Å for aromatic and methyl H atoms, respectively, and constrained to ride on their parent atoms, with *U*_iso_(H) = *xU*_eq_(C), where *x* = 1.5 for methyl H and *x* = 1.2 for aromatic H atoms. One low angle reflection (100) was partially obscured by the beam stop and was omitted from the refinement. The highest peak and deepest hole are located 0.86 and 0.68 Å, respectively, from Pb1.

### Crystal data

**Table:**

[Pb(C_8_H_7_O_2_)_2_(C_6_H_6_N_2_O)]	*F*(000) = 1152
*M**_r_* = 599.60	*D*_x_ = 1.941 Mg m^−^^3^
Monoclinic, *P*2_1_/*c*	Mo *K*α radiation, λ = 0.71073 Å
Hall symbol: -P 2ybc	Cell parameters from 9891 reflections
*a* = 14.1146 (3) Å	θ = 2.4–28.4°
*b* = 7.7431 (2) Å	µ = 8.26 mm^−^^1^
*c* = 19.2165 (4) Å	*T* = 100 K
β = 102.322 (2)°	Plate, colourless
*V* = 2051.81 (8) Å^3^	0.34 × 0.32 × 0.13 mm
*Z* = 4	

### Data collection

**Table:**

Bruker Kappa APEXII CCD area-detector diffractometer	5143 independent reflections
Radiation source: fine-focus sealed tube	4669 reflections with *I* > 2σ(*I*)
graphite	*R*_int_ = 0.030
φ and ω scans	θ_max_ = 28.5°, θ_min_ = 2.2°
Absorption correction: multi-scan (*SADABS*; Bruker, 2005)	*h* = −18→17
*T*_min_ = 0.074, *T*_max_ = 0.342	*k* = −9→10
19461 measured reflections	*l* = −25→25

### Refinement

**Table:**

Refinement on *F*^2^	Primary atom site location: structure-invariant direct methods
Least-squares matrix: full	Secondary atom site location: difference Fourier map
*R*[*F*^2^ > 2σ(*F*^2^)] = 0.020	Hydrogen site location: inferred from neighbouring sites
*wR*(*F*^2^) = 0.050	H atoms treated by a mixture of independent and constrained refinement
*S* = 1.03	*w* = 1/[σ^2^(*F*_o_^2^) + (0.0216*P*)^2^ + 2.0622*P*] where *P* = (*F*_o_^2^ + 2*F*_c_^2^)/3
5143 reflections	(Δ/σ)_max_ = 0.003
281 parameters	Δρ_max_ = 1.08 e Å^−^^3^
0 restraints	Δρ_min_ = −1.03 e Å^−^^3^

### Special details

**Table:**

Geometry. All esds (except the esd in the dihedral angle between two l.s. planes) are estimated using the full covariance matrix. The cell esds are taken into account individually in the estimation of esds in distances, angles and torsion angles; correlations between esds in cell parameters are only used when they are defined by crystal symmetry. An approximate (isotropic) treatment of cell esds is used for estimating esds involving l.s. planes.
Refinement. Refinement of F^2^ against ALL reflections. The weighted R-factor wR and goodness of fit S are based on F^2^, conventional R-factors R are based on F, with F set to zero for negative F^2^. The threshold expression of F^2^ > 2sigma(F^2^) is used only for calculating R-factors(gt) etc. and is not relevant to the choice of reflections for refinement. R-factors based on F^2^ are statistically about twice as large as those based on F, and R- factors based on ALL data will be even larger.

### Fractional atomic coordinates and isotropic or equivalent isotropic displacement parameters (Å^2^)

**Table:**

	*x*	*y*	*z*	*U*_iso_*/*U*_eq_	
Pb1	0.455879 (7)	0.261390 (11)	0.672516 (4)	0.01364 (4)	
O1	0.26579 (14)	0.3383 (2)	0.61028 (9)	0.0203 (4)	
O2	0.38915 (13)	0.5234 (2)	0.62939 (9)	0.0165 (4)	
O3	0.59356 (13)	0.3990 (2)	0.63203 (9)	0.0187 (4)	
O4	0.59623 (14)	0.4377 (2)	0.74604 (9)	0.0198 (4)	
O5	0.42873 (14)	0.1659 (2)	0.53563 (9)	0.0207 (4)	
N1	0.39370 (16)	0.1149 (3)	0.28403 (10)	0.0148 (4)	
N2	0.53688 (17)	0.3091 (3)	0.48605 (12)	0.0185 (4)	
H2A	0.566 (2)	0.351 (4)	0.5262 (17)	0.023 (8)*	
H2B	0.552 (2)	0.347 (4)	0.4480 (17)	0.028 (8)*	
C1	0.29883 (19)	0.4848 (3)	0.60520 (12)	0.0154 (5)	
C2	0.23623 (19)	0.6286 (3)	0.56937 (13)	0.0157 (5)	
C3	0.26372 (19)	0.8006 (3)	0.58144 (13)	0.0176 (5)	
H3	0.3189	0.8277	0.6157	0.021*	
C4	0.2096 (2)	0.9319 (3)	0.54282 (13)	0.0186 (5)	
H4	0.2277	1.0464	0.5524	0.022*	
C5	0.1285 (2)	0.8938 (3)	0.48978 (13)	0.0198 (5)	
C6	0.0983 (2)	0.7224 (4)	0.48013 (15)	0.0227 (6)	
H6	0.0423	0.6958	0.4466	0.027*	
C7	0.1510 (2)	0.5900 (3)	0.52003 (13)	0.0201 (5)	
H7	0.1293	0.4764	0.5138	0.024*	
C8	0.0763 (2)	1.0343 (4)	0.44306 (15)	0.0275 (6)	
H8A	0.0096	1.0025	0.4265	0.041*	
H8B	0.1060	1.0507	0.4030	0.041*	
H8C	0.0800	1.1398	0.4698	0.041*	
C9	0.63657 (19)	0.4482 (3)	0.69346 (12)	0.0156 (5)	
C10	0.73759 (19)	0.5183 (3)	0.70341 (13)	0.0165 (5)	
C11	0.7859 (2)	0.5820 (3)	0.76893 (13)	0.0206 (5)	
H11	0.7547	0.5834	0.8070	0.025*	
C12	0.8796 (2)	0.6431 (3)	0.77827 (14)	0.0246 (6)	
H12	0.9103	0.6877	0.8223	0.030*	
C13	0.9291 (2)	0.6390 (3)	0.72244 (15)	0.0230 (6)	
C14	0.8792 (2)	0.5788 (3)	0.65661 (14)	0.0227 (6)	
H14	0.9099	0.5787	0.6184	0.027*	
C15	0.7850 (2)	0.5191 (3)	0.64661 (13)	0.0195 (5)	
H15	0.7531	0.4794	0.6021	0.023*	
C16	1.0339 (2)	0.6892 (4)	0.73324 (19)	0.0350 (7)	
H16A	1.0436	0.7559	0.6932	0.053*	
H16B	1.0733	0.5870	0.7375	0.053*	
H16C	1.0520	0.7568	0.7759	0.053*	
C17	0.43980 (18)	0.1755 (3)	0.34748 (12)	0.0138 (5)	
H17	0.4933	0.2468	0.3494	0.017*	
C18	0.41142 (18)	0.1368 (3)	0.41091 (12)	0.0137 (5)	
C19	0.33353 (19)	0.0266 (3)	0.40765 (13)	0.0181 (5)	
H19	0.3133	−0.0039	0.4490	0.022*	
C20	0.2860 (2)	−0.0379 (3)	0.34251 (13)	0.0199 (5)	
H20	0.2337	−0.1125	0.3393	0.024*	
C21	0.31782 (19)	0.0106 (3)	0.28202 (13)	0.0173 (5)	
H21	0.2849	−0.0311	0.2381	0.021*	
C22	0.46060 (19)	0.2068 (3)	0.48252 (13)	0.0146 (5)	

### Atomic displacement parameters (Å^2^)

**Table:**

	*U*^11^	*U*^22^	*U*^33^	*U*^12^	*U*^13^	*U*^23^
Pb1	0.01661 (6)	0.01381 (6)	0.01057 (5)	0.00062 (3)	0.00306 (4)	0.00089 (3)
O1	0.0239 (11)	0.0159 (9)	0.0209 (9)	−0.0031 (7)	0.0044 (8)	0.0029 (7)
O2	0.0174 (10)	0.0166 (9)	0.0150 (8)	−0.0008 (7)	0.0023 (7)	0.0036 (6)
O3	0.0175 (10)	0.0249 (10)	0.0129 (8)	−0.0021 (7)	0.0018 (7)	−0.0025 (7)
O4	0.0211 (10)	0.0244 (10)	0.0144 (8)	0.0013 (7)	0.0051 (7)	−0.0013 (7)
O5	0.0240 (11)	0.0265 (10)	0.0129 (8)	−0.0046 (8)	0.0071 (7)	−0.0005 (7)
N1	0.0165 (11)	0.0153 (10)	0.0124 (9)	0.0012 (8)	0.0028 (8)	−0.0010 (7)
N2	0.0227 (12)	0.0222 (11)	0.0110 (10)	−0.0050 (9)	0.0046 (9)	−0.0013 (9)
C1	0.0197 (14)	0.0187 (12)	0.0086 (10)	−0.0009 (9)	0.0045 (9)	−0.0008 (8)
C2	0.0160 (13)	0.0183 (12)	0.0142 (11)	−0.0002 (9)	0.0061 (10)	0.0008 (9)
C3	0.0169 (13)	0.0197 (12)	0.0164 (12)	−0.0023 (10)	0.0037 (10)	−0.0008 (10)
C4	0.0226 (15)	0.0160 (12)	0.0187 (12)	0.0002 (10)	0.0079 (11)	0.0017 (9)
C5	0.0206 (14)	0.0216 (13)	0.0190 (12)	0.0046 (10)	0.0084 (11)	0.0022 (10)
C6	0.0177 (14)	0.0254 (14)	0.0229 (14)	0.0031 (10)	−0.0006 (11)	−0.0025 (10)
C7	0.0188 (14)	0.0196 (13)	0.0207 (12)	−0.0011 (10)	0.0016 (11)	−0.0002 (10)
C8	0.0293 (17)	0.0268 (15)	0.0265 (14)	0.0090 (12)	0.0065 (12)	0.0062 (11)
C9	0.0174 (13)	0.0152 (12)	0.0139 (11)	0.0038 (9)	0.0029 (10)	0.0002 (9)
C10	0.0171 (14)	0.0145 (12)	0.0159 (11)	0.0027 (9)	−0.0008 (10)	0.0005 (9)
C11	0.0221 (15)	0.0212 (13)	0.0171 (12)	0.0050 (10)	0.0010 (10)	−0.0021 (10)
C12	0.0246 (16)	0.0228 (14)	0.0210 (13)	0.0019 (11)	−0.0073 (11)	−0.0050 (10)
C13	0.0208 (15)	0.0138 (12)	0.0308 (14)	0.0003 (10)	−0.0028 (11)	0.0003 (10)
C14	0.0226 (15)	0.0224 (14)	0.0237 (13)	−0.0006 (11)	0.0061 (11)	0.0026 (10)
C15	0.0192 (14)	0.0213 (13)	0.0171 (12)	−0.0027 (10)	0.0021 (10)	−0.0005 (9)
C16	0.0229 (17)	0.0277 (16)	0.0500 (19)	−0.0039 (13)	−0.0019 (14)	0.0004 (14)
C17	0.0139 (12)	0.0131 (12)	0.0144 (11)	−0.0002 (9)	0.0034 (9)	0.0002 (8)
C18	0.0151 (13)	0.0135 (11)	0.0120 (11)	0.0022 (9)	0.0020 (9)	0.0003 (8)
C19	0.0179 (14)	0.0232 (13)	0.0147 (11)	−0.0011 (10)	0.0071 (10)	0.0017 (9)
C20	0.0165 (14)	0.0242 (14)	0.0185 (12)	−0.0061 (10)	0.0025 (10)	0.0018 (10)
C21	0.0168 (14)	0.0213 (13)	0.0126 (11)	−0.0010 (10)	0.0004 (10)	−0.0009 (9)
C22	0.0172 (13)	0.0141 (11)	0.0127 (11)	0.0018 (9)	0.0034 (9)	−0.0003 (9)

### Geometric parameters (Å, °)

**Table:**

Pb1—O1	2.7594 (19)	C9—O3	1.265 (3)
Pb1—O2	2.3141 (17)	C9—C10	1.499 (4)
Pb1—O3	2.4824 (18)	C10—C11	1.388 (3)
Pb1—O4	2.5672 (19)	C10—C15	1.397 (4)
Pb1—O5	2.6800 (16)	C11—C12	1.380 (4)
Pb1—N1^i^	2.661 (2)	C11—H11	0.93
O1—C1	1.239 (3)	C12—H12	0.93
O2—C1	1.295 (3)	C13—C12	1.400 (4)
O4—C9	1.264 (3)	C13—C14	1.391 (4)
N1—Pb1^ii^	2.661 (2)	C13—C16	1.500 (4)
N2—C22	1.327 (4)	C14—H14	0.93
N2—H2A	0.86 (3)	C15—C14	1.382 (4)
N2—H2B	0.85 (3)	C15—H15	0.93
C1—C2	1.495 (3)	C16—H16A	0.96
C2—C7	1.396 (4)	C16—H16B	0.96
C3—C2	1.393 (4)	C16—H16C	0.96
C3—C4	1.388 (4)	C17—N1	1.338 (3)
C3—H3	0.93	C17—C18	1.394 (3)
C4—C5	1.393 (4)	C17—H17	0.93
C4—H4	0.93	C18—C19	1.383 (3)
C5—C8	1.500 (4)	C18—C22	1.504 (3)
C6—C5	1.394 (4)	C19—C20	1.381 (3)
C6—C7	1.396 (4)	C19—H19	0.93
C6—H6	0.93	C20—H20	0.93
C7—H7	0.93	C21—N1	1.335 (3)
C8—H8A	0.96	C21—C20	1.384 (3)
C8—H8B	0.96	C21—H21	0.93
C8—H8C	0.96	C22—O5	1.241 (3)
			
O2—Pb1—O3	78.32 (6)	C5—C8—H8C	109.5
O2—Pb1—O4	86.45 (6)	H8A—C8—H8B	109.5
O3—Pb1—O4	51.71 (5)	H8A—C8—H8C	109.5
O2—Pb1—N1^i^	78.11 (6)	H8B—C8—H8C	109.5
O3—Pb1—N1^i^	120.84 (6)	O4—C9—O3	121.2 (2)
O4—Pb1—N1^i^	73.37 (6)	O4—C9—C10	120.0 (2)
O2—Pb1—O5	85.89 (6)	O3—C9—C10	118.8 (2)
O3—Pb1—O5	76.61 (5)	C11—C10—C15	118.9 (3)
O4—Pb1—O5	128.23 (5)	C11—C10—C9	120.9 (2)
N1^i^—Pb1—O5	152.50 (6)	C15—C10—C9	120.2 (2)
O2—Pb1—O1	51.09 (6)	C10—C11—H11	119.6
O3—Pb1—O1	121.69 (5)	C12—C11—C10	120.8 (3)
O4—Pb1—O1	133.51 (5)	C12—C11—H11	119.6
N1^i^—Pb1—O1	79.28 (6)	C11—C12—C13	120.9 (2)
O5—Pb1—O1	73.26 (6)	C11—C12—H12	119.5
C1—O1—Pb1	83.35 (15)	C13—C12—H12	119.5
C1—O2—Pb1	102.76 (14)	C12—C13—C16	121.8 (3)
C9—O3—Pb1	95.09 (15)	C14—C13—C12	117.7 (3)
C9—O4—Pb1	91.18 (15)	C14—C13—C16	120.4 (3)
C22—O5—Pb1	137.38 (17)	C13—C14—H14	119.2
C21—N1—C17	118.0 (2)	C15—C14—C13	121.6 (3)
C21—N1—Pb1^ii^	126.49 (15)	C15—C14—H14	119.2
C17—N1—Pb1^ii^	115.32 (16)	C10—C15—H15	120.0
C22—N2—H2A	120 (2)	C14—C15—C10	120.0 (2)
C22—N2—H2B	120 (2)	C14—C15—H15	120.0
H2A—N2—H2B	119 (3)	C13—C16—H16A	109.5
O1—C1—O2	122.8 (2)	C13—C16—H16B	109.5
O1—C1—C2	121.5 (2)	C13—C16—H16C	109.5
O2—C1—C2	115.7 (2)	H16A—C16—H16B	109.5
C3—C2—C1	121.3 (2)	H16A—C16—H16C	109.5
C3—C2—C7	119.2 (2)	H16B—C16—H16C	109.5
C7—C2—C1	119.5 (2)	N1—C17—C18	123.1 (2)
C4—C3—C2	120.6 (2)	N1—C17—H17	118.4
C4—C3—H3	119.7	C18—C17—H17	118.4
C2—C3—H3	119.7	C17—C18—C22	124.0 (2)
C3—C4—C5	120.6 (2)	C19—C18—C17	117.8 (2)
C3—C4—H4	119.7	C19—C18—C22	118.1 (2)
C5—C4—H4	119.7	C18—C19—H19	120.3
C4—C5—C6	118.6 (2)	C20—C19—C18	119.5 (2)
C4—C5—C8	120.4 (2)	C20—C19—H19	120.3
C6—C5—C8	120.9 (3)	C19—C20—C21	118.8 (2)
C5—C6—C7	121.0 (3)	C19—C20—H20	120.6
C5—C6—H6	119.5	C21—C20—H20	120.6
C7—C6—H6	119.5	N1—C21—C20	122.8 (2)
C2—C7—H7	120.1	N1—C21—H21	118.6
C6—C7—C2	119.8 (2)	C20—C21—H21	118.6
C6—C7—H7	120.1	O5—C22—N2	122.9 (2)
C5—C8—H8A	109.5	O5—C22—C18	118.9 (2)
C5—C8—H8B	109.5	N2—C22—C18	118.2 (2)
			
O2—Pb1—O1—C1	1.22 (13)	C2—C3—C4—C5	−1.9 (4)
O3—Pb1—O1—C1	−35.24 (15)	C3—C4—C5—C6	5.0 (4)
O4—Pb1—O1—C1	30.06 (16)	C3—C4—C5—C8	−173.6 (2)
O5—Pb1—O1—C1	−97.21 (14)	C7—C6—C5—C4	−3.4 (4)
N1^i^—Pb1—O1—C1	84.52 (14)	C7—C6—C5—C8	175.2 (3)
O1—Pb1—O2—C1	−1.19 (12)	C5—C6—C7—C2	−1.3 (4)
O3—Pb1—O2—C1	147.72 (14)	O4—C9—O3—Pb1	−9.4 (2)
O4—Pb1—O2—C1	−160.67 (14)	C10—C9—O3—Pb1	169.95 (19)
O5—Pb1—O2—C1	70.57 (14)	O3—C9—C10—C11	176.7 (2)
N1^i^—Pb1—O2—C1	−86.93 (14)	O3—C9—C10—C15	−3.8 (4)
O1—Pb1—O3—C9	127.94 (14)	O4—C9—C10—C11	−3.9 (4)
O2—Pb1—O3—C9	99.77 (15)	O4—C9—C10—C15	175.6 (2)
O4—Pb1—O3—C9	5.02 (13)	C9—C10—C11—C12	178.7 (2)
O5—Pb1—O3—C9	−171.72 (15)	C15—C10—C11—C12	−0.8 (4)
N1^i^—Pb1—O3—C9	31.39 (16)	C9—C10—C15—C14	−178.0 (2)
O1—Pb1—O4—C9	−105.01 (15)	C11—C10—C15—C14	1.5 (4)
O2—Pb1—O4—C9	−82.92 (14)	C10—C11—C12—C13	−1.4 (4)
O3—Pb1—O4—C9	−5.01 (13)	C14—C13—C12—C11	2.9 (4)
O5—Pb1—O4—C9	−0.98 (17)	C16—C13—C12—C11	−174.1 (3)
N1^i^—Pb1—O4—C9	−161.56 (15)	C12—C13—C14—C15	−2.2 (4)
O1—Pb1—O5—C22	116.6 (3)	C16—C13—C14—C15	174.9 (3)
O2—Pb1—O5—C22	66.1 (3)	C10—C15—C14—C13	0.0 (4)
O3—Pb1—O5—C22	−12.9 (3)	C18—C17—N1—Pb1^ii^	174.19 (18)
O4—Pb1—O5—C22	−16.1 (3)	C18—C17—N1—C21	−1.0 (4)
N1^i^—Pb1—O5—C22	120.2 (3)	N1—C17—C18—C19	1.9 (4)
Pb1—O1—C1—O2	−2.0 (2)	N1—C17—C18—C22	−178.5 (2)
Pb1—O1—C1—C2	177.1 (2)	C17—C18—C19—C20	−1.2 (4)
Pb1—O2—C1—O1	2.5 (3)	C22—C18—C19—C20	179.1 (2)
Pb1—O2—C1—C2	−176.69 (16)	C17—C18—C22—O5	179.1 (2)
Pb1—O4—C9—O3	9.0 (2)	C17—C18—C22—N2	−1.3 (4)
Pb1—O4—C9—C10	−170.3 (2)	C19—C18—C22—O5	−1.3 (4)
O1—C1—C2—C3	160.7 (2)	C19—C18—C22—N2	178.3 (2)
O1—C1—C2—C7	−22.6 (3)	C18—C19—C20—C21	−0.2 (4)
O2—C1—C2—C3	−20.1 (3)	C20—C21—N1—Pb1^ii^	−175.19 (19)
O2—C1—C2—C7	156.5 (2)	C20—C21—N1—C17	−0.6 (4)
C1—C2—C7—C6	−172.3 (2)	N1—C21—C20—C19	1.2 (4)
C3—C2—C7—C6	4.4 (4)	N2—C22—O5—Pb1	10.7 (4)
C4—C3—C2—C1	173.8 (2)	C18—C22—O5—Pb1	−169.76 (16)
C4—C3—C2—C7	−2.8 (4)		

Symmetry codes: (i) *x*, −*y*+1/2, *z*+1/2; (ii) *x*, −*y*+1/2, *z*−1/2.

### Hydrogen-bond geometry (Å, °)

**Table:**

*D*—H···*A*	*D*—H	H···*A*	*D*···*A*	*D*—H···*A*
N2—H2A···O3	0.86 (3)	2.02 (3)	2.835 (3)	158 (3)
N2—H2B···O2^iii^	0.86 (3)	2.11 (3)	2.946 (3)	167 (3)
C4—H4···O1^iv^	0.93	2.53	3.431 (3)	165
C11—H11···O1^v^	0.93	2.59	3.253 (3)	129
C17—H17···O2^iii^	0.93	2.41	3.317 (3)	166

Symmetry codes: (iii) −*x*+1, −*y*+1, −*z*+1; (iv) *x*, *y*+1, *z*; (v) .
